# Liberia’s First Health Workforce Program Strategy: Reflections and Lessons Learned

**DOI:** 10.5334/aogh.3242

**Published:** 2021-10-08

**Authors:** Bernice Dahn, Lila Kerr, Tej Nuthulaganti, Moses Massaquoi, Marion Subah, Attila Yaman, Chelsea M. Plyler, Corrado Cancedda, Roseda E. Marshall, Regan H. Marsh

**Affiliations:** 1 College of Health Sciences, University of Liberia, LR; 2 Brigham and Women’s Hospital, Partners In Health, US; 3 Clinton Health Access Initiative, US; 4 Clinton Health Access Initiative, LR; 5 Last Mile Health, LR; 6 Yale School of Medicine, US; 7 Center for Global Health, Perelman School of Medicine, University of Pennsylvania, US; 8 Acting Chair, Faculty of Pediatrics, LCPS/JFKMC, LR

## Abstract

Following civil war and the Ebola epidemic, Liberia’s health workforce was devastated, essential health services and primary care were disrupted, and health outcomes for maternal and child mortality were amongst the worst in the world. To reverse these trends, the government of Liberia developed the *Health Workforce Program (HWP) Strategy 2015–2021*. With the goal of building a resilient and responsive health system to ensure access to essential services and the ability to respond to future crises, this strategy aimed to add 6,000 new professionals to the workforce. In the context of the COVID-19 pandemic, we share lessons learned from the program’s development and first years of implementation.

## Introduction

The estimated global shortage of 18 million health professionals threatens global progress towards universal health coverage [1]. This shortage is neither evenly nor equitably distributed: relative to population, sub-Saharan Africa bears the highest burden of disease, experiences the most severe shortage of health workers, and is the only region where the shortage is expected to increase by 2030 [1,2].

In the last decade, the global community has recognized the importance of workforce to improving health outcomes. Setting the agenda, the World Health Organization (WHO) Region for Africa adopted the *Regional Road Map for Scaling Up the Health Workforce from 2012 to 2025* [3]. At the same time, traditionally “vertically-oriented” funders, such as the Global Fund to Fight AIDS, Tuberculosis, and Malaria and the President’s Emergency Plan for AIDS Relief (PEPFAR) began supporting health workforce interventions [4,5]. Pivotal early investments from PEPFAR—the Medical Education Partner Initiative (MEPI) and Nursing Education Partnership Initiative (NEPI)—strengthened the number and quality of clinicians in sub-Saharan Africa [6]. This was followed by Rwanda’s ambitious Human Resources for Health program, which launched 17 training programs for 5,600 health workers and serves as an international benchmark [5].

In Liberia, following a series of humanitarian crises including civil war and the Ebola outbreak, the health workforce shortage required urgent action. In this context, the Ministry of Health (MOH) quickly moved to develop its first national health workforce strategy. We share lessons learned from its development and first years of implementation.

## Liberian Context

Over a decade of civil war (1989–1997 and 1999–2003) devastated Liberia’s health system. Of 293 public health facilities, 242 were destroyed and health outcomes plummeted. At the war’s conclusion, under-five mortality and maternal mortality were amongst the worst globally [7]. The health workforce was depleted due to migration and death. By 1998, the total public health personnel had fallen from 3, 526 to 1, 396 [8], with the number of physicians declining to fewer than 30 [9]. Specialist physician and nurses were in critical need [10].

Exacerbating the crisis, training of health workers had almost entirely stalled, with many schools forced to close during the conflict. After the war, there was tremendous need to rebuild infrastructure, increase the number of faculty, accredit institutions, and update curricula, which had not been revised since the start of the war [8,11].

Facing this challenge, the government launched the *Emergency Human Resources for Health Plan for 2007–2011*, with the goal of rapidly increasing the number of health workers [12]. To ensure sustainability, the MOH prioritized creating a health workforce program, but progress was interrupted by the Ebola outbreak.

Between 2014–2016, Liberia suffered 10,678 cases and 4,810 deaths due to Ebola Virus Disease (EVD) [13], compounding the health workforce challenge with 375 health worker infections and 189 deaths [14]. Nationally, 8% of physicians, nurses, and midwives died [15]. By 2016, the workforce density was 11 health workers per 10,000 population, compared to the recommended 23 per 10, 000 [16]. Grievously, health educators and leaders died, including the renowned heads of the Internal Medicine and Emergency services at the national academic medical center [17].

Service delivery was impacted across all sectors, with decreases in malaria treatment, immunizations, antenatal and postnatal care, facility-based deliveries, and HIV and TB treatment [18,19,20]. The maternal mortality rate worsened from 640 to 1,347 per 100,000 live births, and the under-five mortality rate increased from 71 to 91 per 1,000 live births [15]. As the outbreak subsided, it was imperative to address the crisis that was Liberia’s health workforce.

## Development of the Health Workforce Program Strategy

**Figure 1:**
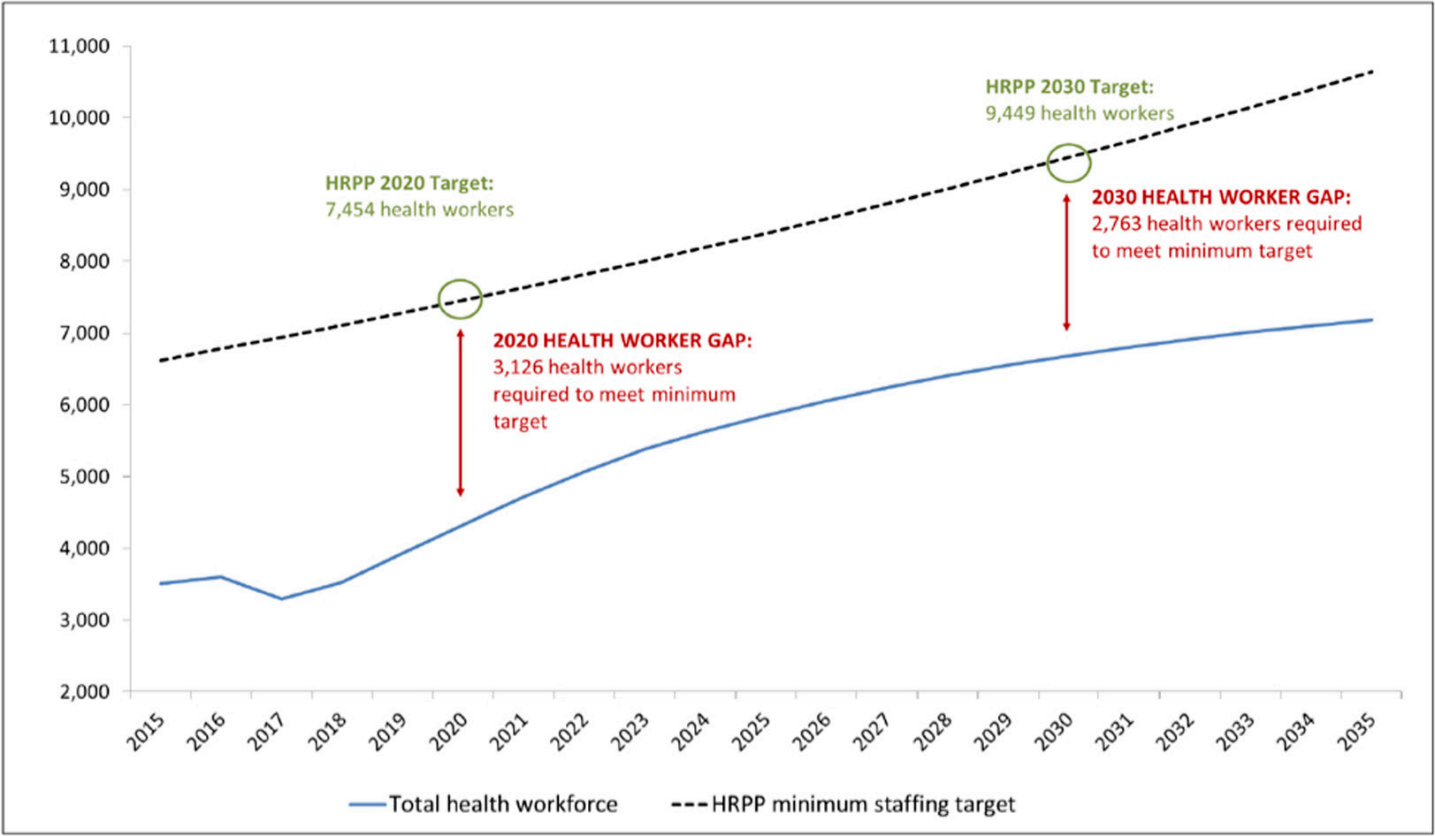
Based on the 2014 Health Workforce Training Institutions Assessment, at projected rates of production and attrition, the gap in Liberia’s Health Workforce would continue to grow, falling short of the Human Resources Policy and Plan 2011–2022 (HRPP) targets.

Early health workforce activities after the civil war included launching physician residency training programs and updating accreditation standards for nursing and midwifery training, as well as limited faculty development initiatives. Despite this, the 2014 *Health Workforce Training Institutions Assessment* showed that output was not keeping pace with population growth and health worker attrition (***Figure 1***), and quality of training was a concern for all cadres [21].

To accelerate strategic investment to reach its targets, the government developed the *Health Workforce Program (HWP) Strategy 2015–2021* to improve the quality, quantity, and skill diversity of the national health workforce [22]. Incorporating lessons from the Ebola response, the HWP targeted strengthening general and specialist physicians, general and specialized nurses, and midwives, and formalizing two new cadres—health managers and community health assistants.

For physician training, increasing the number of general practitioners was the top priority, and condensing the nine-year training pathway was identified as key to reducing attrition. The HWP also called for increased specialty training, including strengthening the new residency training programs, and, later, creating fellowship programs. Study abroad programs were incorporated to train educators and health professionals in priority specialties unavailable in Liberia.

Nursing and midwifery required distinct approaches: whereas there was an acute shortage of midwives to address, the graduation rate for nurses was sufficient given a post-war proliferation of nursing schools; though, consequently, quality remained a concern. For both cadres, there was a need to upgrade existing certificate-level clinicians by offering “bridging” pathways to become a Registered Nurse or Midwife or to earn a Bachelor’s degree [10]. The HWP also called for nurse specialist training, including a Nurse Anesthetist program and a Master of Nurse and Midwifery Education.

Across all cadres, the HWP sought to strengthen the quality of training through curricula revision, investments in training infrastructure, and faculty development. Proposed infrastructure investments targeted 10 training institutions for upgrades in skills labs, classrooms, and student dormitories and also identified three priority teaching hospitals to be rebuilt or expanded to improve the clinical training environment.

The HWP aimed to add 6,000 health workers over baseline. To achieve these goals, 75 visiting faculty were to be recruited to fill essential teaching and service gaps, while building long-term national faculty. Visiting faculty would serve as peer-mentors and were selected preferentially from the West African College of Physicians and Surgeons. International academic partners were to provide institutional resources and to provide technical assistance.

The HWP was projected to cost 15–30 million USD annually over the 7-year program, plus an additional 45 million USD in one-time infrastructure investments. Mobilizing external financial investments was imperative, but in the context of Ebola, there was broad agreement from funders that building resilient health systems was critical, and the HWP was an attractive investment. A wide range of funders, including the World Bank, the Global Fund, PEPFAR, and U.S. Agency for International Development (USAID) agreed to support the program.

## Lessons Learned

While the total impact of the HWP will become clear following its conclusion, there are lessons to be learned for academic institutions, government leaders, policy makers, and financing partners on how to successfully develop and manage such a large-scale program, now viewed through the lens of another pandemic, COVID-19.

### Health workforce investments have been critical to improving the resilience and responsiveness of the health system

In the HWP’s sixth year, Liberia registered its first COVID-19 cases. Building on lessons from Ebola and buttressed by the strengthened training programs, leadership quickly pivoted at national and institutional levels to respond to the pandemic.

Prior to the Ebola crisis and the HWP, many Liberian physicians had not been comprehensively trained in infectious diseases due to the lack of qualified specialists. As part of the HWP, medical students and resident training had prioritized this specialty, essential to the nimble COVID-19 response. Trained health managers and leaders at Liberian health facilities quickly implemented infection prevention and control protocols to ensure staff and patient safety and to avoid disruption to essential services. Training programs adapted to social distancing guidelines by shifting coursework online and implementing innovative models for remote supervision.

### Continuous national leadership and active coordination are critical for mobilizing and aligning resources for large-scale implementation

The development of a robust, government-approved strategy outlining specific interventions and expected impact has been critical to mobilizing resources and aligning partners. The most senior levels of government supported the HWP development, and advocacy efforts resulted in roughly 80 million USD in funding, nearly half of the amount required.

No single organization was able to fund the program alone, thus multiple donors were secured through a coordination forum. This complex network of stakeholders has required ongoing management by national leaders—at one point, there were at least 8 funding agencies investing through at least 13 implementing partners, some of whom were receiving funding from multiple sources and many of whom were working with the same Liberian institutions. As implementation has progressed, working directly with the local institutions has strengthened partner coordination and success, even as national elections resulted in governmental leadership changes.

### Training faculty to lead training programs for future generations is key to sustainability but takes time and is optimized with investments in clinical infrastructure

Misalignment of stakeholder implementation timelines has challenged the full potential of the HWP. As recently noted in a PEPFAR-commissioned study of the Rwanda HRH Program “…the duration required to build institutional capacity is on the order of decades, whereas the [Rwanda] HRH Program, as laid out in the 2011 proposal, was planned for 9 years and faced a significant drop in funding following the cessation of PEPFAR investment after 5 years” [23].

Similarly, in Liberia, the seven-year program was designed to meet the need for training programs over multiple years; whereas, funder commitments were shorter, often with yearly contract renewals. Of the at least eight funding agencies supporting the HWP, none committed upfront to the full seven-year implementation envisioned by the MOH. Health professions education takes several years to graduate a class of students and then to identify and train a subset in academic pathways to serve as faculty. The lack of multiyear funding commitments left some training programs with interruptions in visiting faculty and programmatic support. Insufficient planning and lead time, associated with one-year renewed funding windows, constrained visiting faculty recruitment and rehabilitation of key infrastructure. Additionally, as donor governments shifted their priorities for international development, Liberian implementing partners were required to shift their programmatic work, at times in ways that competed with the HWP priorities. While common practice amongst funding agencies, this posed a significant burden to mobilize, negotiate, and renew funding, impeding progress.

Additionally, at times, funder-driven priorities resulted in uneven investment across the HWP, with donors funding technical assistance and training without complementary health infrastructure. However, health workforce training is inseparable from the clinical service delivery environment. In one example, a visiting faculty pathologist found no pathology lab available to provide service or train students. Ultimately, governmental advocacy resulted in funding for this domain. However overall, while notable infrastructure progress has occurred, serious gaps persist in biomedical and laboratory equipment, as well as electronic health and laboratory records.

### A focus on institutional capacity building is necessary to ensure sustainability of programming at training institutions and teaching hospitals

To ensure the sustainability of investments, the HWP provided a vision for training the next generation of Liberian faculty who would ultimately lead these institutions, ensuring that international academic partnerships prioritized long-term capacity building. Academic partners recruited visiting faculty from West African institutions to ensure trainees received high-quality, contextually relevant training in accordance with regional standards.

The HWP implementation has underscored the importance of administrative support structures. A study-tour to an international academic partner revealed that their school had five administrative staff for every faculty member; whereas, Liberian institutions had far fewer. In future workforce programs, administrative capacity at local institutions should be prioritized to achieve systems-level successes and sustainability.

Lastly, the HWP showed that quality could not be maintained without setting and enforcing standards through accreditation and regulatory agencies. Major investments were focused on strengthening the Liberia Board of Nursing and Midwifery and the newly formed Liberia College of Physicians and Surgeons. These regulatory bodies will ensure quality of health workers and training programs for the long-term.

## Conclusion

The HWP was born out of the Government of Liberia’s long-standing recognition of health workers’ role in ensuring equitable access to healthcare for all. Now, in the face of another pandemic, the centrality of the health workforce to the delivery of essential services is even more clear, as these investments in human resources have paid dividends, enhancing the response while ensuring continued primary care delivery and health worker training. Crises will continue to arise, requiring leadership by governments and health professionals alike. Partnerships and investment to capacitate health professionals at every level of the system remains critical. As Liberia continues to strengthen its health sector through COVID-19, we hope that the lessons learned can serve as a model for the next phase of health workforce training here and in other countries.
